# The Potential of Cultivated Mushrooms as Salt Substitutes in Meat Products

**DOI:** 10.3390/foods14060977

**Published:** 2025-03-13

**Authors:** Juana Fernández-López, Manuel Viuda-Martos, Carmen Botella-Martínez, Clara Muñoz-Bas, Patricia Bermúdez-Gómez, Raquel Lucas-González, José Ángel Pérez-Álvarez

**Affiliations:** 1 IPOA Research Group, Institute for Agri-Food and Agri-Environmental Research and Innovation (CIAGRO-UMH), Miguel Hernández University, 03312 Orihuela, Alicante, Spain; mviuda@umh.es (M.V.-M.); c.botella@umh.es (C.B.-M.); clara.munozb@umh.es (C.M.-B.); raquel.lucasg@umh.es (R.L.-G.); ja.perez@umh.es (J.Á.P.-Á.); 2 Mushroom Technological Research Center of La Rioja (CTICH), Carretera Calahorra, km 4, 26560 Autol, La Rioja, Spain; patricia.bermudez@goumh.umh.es

**Keywords:** salt-reduced, meat products, umami flavor, sensory properties, natural ingredients, health benefits

## Abstract

This study reviews the feasibility of using cultivated mushrooms in the development of salt-reduced meat products. For this purpose, it is important to know the role of salt in meat products in order to develop viable strategies for its substitution. In addition, mushroom types and properties (composition, nutritional value, umami content, etc.) and examples of successful application as salt substitutes in meat products are addressed. Salt has important roles in meat product processing, mainly affecting its technological, antimicrobial, and sensory properties. Therefore, the different strategies that have been studied (meat product reformulation and technological advances) with the aim of reducing its content have to address these effects. The application of mushrooms as a salt substitute shows several advantages mainly related to the fact that mushrooms are a natural ingredient with a very healthy nutritional composition (rich in protein and dietary fiber but low in fat and sodium) and, from an economic and sustainable cultivation perspective, aligns well with current trends in food production and consumption. Salt substitutions of 50% have been achieved, mainly in fresh meat products (hamburgers) and heat-treated meat products (sausages, pâté, roast meat, etc.), with minimal physicochemical and sensory modifications of the final product. The meat industry could benefit from incorporating cultivated mushrooms as a salt-reducing ingredient, especially in the development of reduced salt meat products with a quality comparable to or superior to traditional products. The optimization of processes for their integration in the formulation of meat products should be the trend to ensure their viability.

## 1. Introduction

There is much evidence linking excessive salt consumption with cardiovascular health problems. Studies on the eating habits of the Spanish population reveal a salt intake far in excess of expert recommendations: an average of 10 g of salt is ingested per day when recommendations call for no more than 5 g per day [1]. It is also estimated that 70–80% of salt intake comes from processed foods, especially bread and meat products [2].

Therefore, the development of low-salt meat products is one of the current challenges facing the meat industry. In addition, the reformulated meat products must comply with standards regulating the nutrition and health claims of food [3]. In the case of products whose salt or sodium content has been modified, they may only include the claim “low sodium/salt” on the label if the product contains no more than 0.12 g of sodium or the equivalent value of salt per 100 g. Taking into account that 1 gram of salt contains about 390 milligrams of sodium, such indication shall ensure that it does not contain more than 0.04 g of sodium or the equivalent value of salt per 100 g. Products labeled as “very low sodium/salt” shall not contain more than 0.04 g of sodium or the equivalent value of salt, and those labeled as “sodium-free or salt-free” shall not contain more than 0.005 g of sodium or the equivalent value of salt per 100 g. In the development of low-salt meat products, consumer expectations regarding their preference for “clean label” foods, their concerns about the use of artificial additives, and the sustainability of food production processes (according to the Sustainable Development Goals (SDGs) of the UN 2030 Agenda) [4], should also be taken into account. In this line, the salt replacement should be made with natural compounds rather than other additives and also from sustainable food production. In this regard, the use of cultivated mushrooms as salt substitutes in meat products would meet these requirements (natural and sustainably grown products) [5]. In addition to this, mushrooms are notable for their nutritional value, the content of phenolic compounds (antioxidants), and compounds responsible for the umami taste, which make them a good substitute for salt. Mushrooms are able to provide flavor and aroma, often even categorized as “meat aroma” [6,7,8], which would improve the sensory characteristics of low-salt meat products [6,7].

This review explores the role of cultivated mushrooms in reducing salt content in meat products, focusing on their composition, functional properties, and impact on sensory and physicochemical characteristics.

## 2. Mushrooms: Composition, Functional Properties, and Food Applications

### 2.1. Composition and Functional Properties of Edible Mushrooms

Although the consumption of mushrooms has been prevalent in society for an extended period, recent years have witnessed a notable increase in interest and research regarding the utilization of edible mushrooms as food ingredients in food production processes. The rising demand for plant-based products, alongside the growing focus on health-promoting and functional foods, are likely primary drivers of this trend. Additionally, the sustainability associated with mushroom cultivation [9,10,11], which is regarded as a straightforward and economically viable process, plays a significant role. This process can be conducted using agro-industrial byproducts such as cereals, cotton, fruits, vegetables, sawdust, and leaves [5,12,13,14,15,16], thereby offering an opportunity for generating supplementary income for local producers [9,10,15].

**Figure 1:**
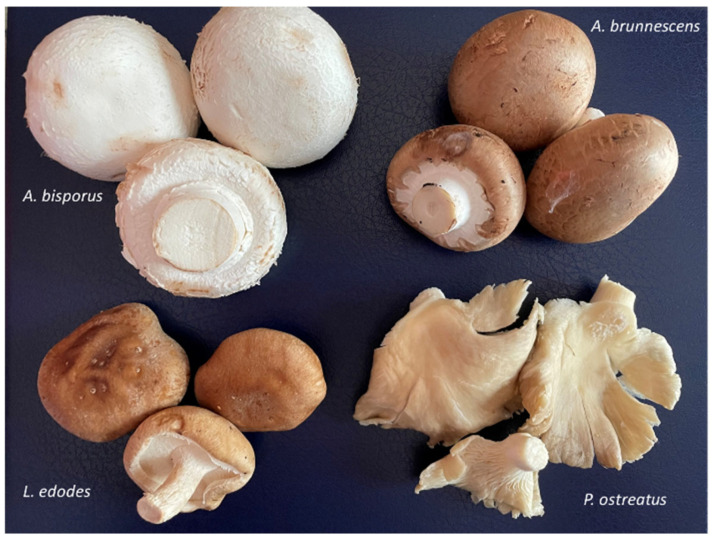
Main mushrooms cultivated in Spain.

Traditionally, the popularity of mushroom consumption has been mainly attributed to their aroma and taste, but in recent years, their high nutritional value and bioactive compound content have also been highlighted [16,17,18,19]. These properties of mushrooms vary greatly depending on the species. Although more than 2000 species of mushrooms have been identified as safe and therefore suitable for consumption or medicinal use [20], in Spain, for example, legislation [21] allows only 34 cultivated species to be marketed fresh, the majority of species are white mushroom (*A. bisporus*), followed by oyster mushroom (*Pleurotus ostreatus*), brown mushroom (*Agaricus brunnescens*), Chinese or shiitake mushroom (*Lentinula edodes*), and truffle (*Tuber melanosporum*, mainly preserved) [22] (Figure 1).

Although the composition varies greatly between species, mushrooms are considered to contain 90% water and, of the dry extract (ES approx. 10%), the major components are carbohydrates (35–40 g/100 g ES), dietary fiber (25–30 g/100 g ES), protein (20–25 g/100 g ES), and ash (5–12 g/100 g ES) [23,24,25,26]. Table 1 shows the chemical composition of the most widely consumed edible mushrooms in Spain.

In general, mushrooms are nutritionally low in energy (values below 40–50 kcal/100 g fresh weight), fat (0.2–0.3 g/100 g fresh weight), and sodium (less than 5 mg/100 g fresh weight), but high in protein, carbohydrates, and dietary fiber [18,27,28]. All essential amino acids are present in mushrooms, and they contain a higher proportion of unsaturated than saturated fatty acids [29]. In reference to dietary fiber, it is mainly insoluble (mainly chitin, characteristic of mushrooms, and some beta-glucans) [30]. In addition, mushrooms have various vitamins (mostly calciferol (vit. D), ascorbic acid (vit. C), niacin (vit. B3), riboflavin (vit. B2), cobalamin (vit. B12), and pantothenic acid (vit. B5)), minerals (mainly potassium, phosphorus, zinc, and selenium) and other minority compounds such as carotenoids, phenolic compounds (such as gallic acid, protocatechuic acid, catechin, caffeic acid, ferulic acid, and myricetin), tocopherols, flavonoids, terpenes, steroids, ergothioneine, etc., which contribute to their functional properties [31,32,33]. Many of these compounds have been linked to health and disease prevention properties, attributed to the biological properties of these compounds, including antioxidant, antimicrobial, anti-inflammatory, and antihypercholesterolemic properties [34,35,36]. Prebiotic properties have also been described for the consumption of certain edible mushrooms, mainly attributed to their content of certain polysaccharides such as chitin, hemicellulose, beta- and alpha-glucans, galactans, etc. [37,38].

In addition, one of the most interesting features of mushrooms is their content of umami-like flavorings such as glutamic and aspartic free amino acids and 5′-ribonucleotides (such as 5′-guanosine monophosphate (5′-GMP), 5′-inosine monophosphate (5′-IMP), and 5′-xanthosine monophosphate (5′-XMP)) [39,40]. Different peptides (glutamic-containing) with unique flavoring properties have also been found in mushrooms and may even interact with other volatile compounds, influencing the final aroma and flavor of foods [8,41]. For this reason, mushrooms (in spite of their low salt content) contribute to the salty taste of meat products, reducing the need to add salt to those strictly necessary to develop their important technological effects.

The composition of mushrooms varies considerably depending not only on the species but also on intra-species variability (stage of development and pre- and post-harvest conditions), part of the mushroom, geographical location, environmental conditions, and conditions under which they are preserved after harvesting [42,43,44].

### 2.2. Applications of Cultivated Mushrooms as Substitutes for Food Ingredients and/or Additives

In food production processes, and always with the aim of developing healthier foods, edible mushrooms have been used as substitutes for different food ingredients such as animal proteins, animal fats, or even cereal flours. In the case of substituting food additives, they have been used as substitutes for salt or phosphates, as emulsifiers, texturizers, preservatives, etc.

As mentioned above, edible mushrooms can be considered an important source of good quality protein, even better than other plant protein sources (with a higher content of essential amino acids). For this reason, they have been used as substitutes for animal protein (mainly meat) in the development of meat foods for flexitarians and/or vegans [18]. This high protein content, together with their dietary fiber content and low fat content (predominantly unsaturated fatty acids), make them an optimal ingredient for emerging dietary trends that emphasize the consumption of healthier foods. These foods are often characterized by a reduced reliance on animal-derived proteins or are exclusively based on plant-derived sources.

In this regard, there are studies in which flour from different species of edible mushrooms (*Agaricus bisporus*, *Lentinula edodes*, *Pleurotus sapidus*, or *Flammulina velutipes*) has been used as a substitute for chicken, beef, or pork in the preparation of meat products such as hamburgers, sausages, nuggets, or even taco fillings [45,46,47]. In all of them, partial substitutions (up to 45%) of meat by mushroom flour have been achieved without negatively affecting the sensory properties of the processed products. In addition, this substitution also managed to improve the nutritional (higher fiber content) and functional (higher content of phenolic compounds, greater antioxidant capacity, greater oxidative stability, etc.) properties in the reformulated products. Even lately, there is a preference for using mushroom proteins rather than other non-meat proteins (soya, beans, peas, etc.) for the development of plant-based meat products. The main reasons for that are (i) the umami flavor of mushrooms is preferred over the beany flavor of vegetal protein, (ii) the filamentous and fibrous proteins of mushrooms give the product a texture comparable to meat, (iii) their nutraceutical content provides several health benefits to consumers, and (iv) their antimicrobial components improve the shelf life of the meat analogs [8].

Edible mushrooms have also been successfully used as fat substitutes in meat products, achieving a significant reduction in their calorie content. In this case, there are studies on the use of different edible mushrooms (*Pleurotus eryngii*, *Pleurotus ostreatus*, and *Agaricus bisporus*) as a substitute for pork fat in sausages such as frankfurters, pâté and hamburgers [46,48,49,50]. In some cases, in addition to their application in the form of flour, other formats (raw, boiled, fried, etc.) were tested. In all of them, in addition to the expected final reduction in the fat content of the product, increases in protein and dietary fiber were observed, as well as a greater water retention capacity, which contributed to a reduction in water losses after cooking. In some cases, a reduction of up to 50% of animal fat was achieved without affecting sensory properties.

Due to their easy preparation and preservation in the form of “mushroom powder or flour” after drying and chopping, their use has also been studied as a substitute for flour (mainly cereals such as wheat, but also rice flour) in the preparation of derived products such as pasta, bakery products, snacks, etc. In this case, flours from different species (*Agaricus bisporus*, *Lentinula edodes*, *Boletus edulis*, or *Calocybe indica*, among others) have been used in different products such as noodles, biscuits, cookies, etc. [47,51,52,53,54,55,56,57]. In pasta, up to 10% of wheat flour (semolina) was replaced, resulting in products with some modifications in their textural properties (higher firmness and cooking losses or color modifications, among others) but with higher antioxidant capacity and lower glycaemic index. Similar 5–15% substitutions were considered acceptable in the case of biscuits and cookies without affecting the sensory properties compared to the control. In all of them, their nutritional improvements, concerning their increased protein and dietary fiber, were also considered positive.

In reference to the application of mushroom flour as a substitute for different food additives, the most common use has been as a salt substitute, especially in meat products (which will be discussed in more detail in point 4). Moreover, there are also studies on their possible application as substitutes for phosphates or nitrites in meat products, as binders to replace egg white in meat products for vegans, and even as substitutes for monosodium glutamate (flavoring) in different foods [16,18,58]. These applications would reduce the negative aspects associated with the presence of additives and certain chemical compounds in foods, contributing to “clean label” formulations, which are widely accepted by consumers today.

The role of phosphates in meat products is mainly due to their action on increasing the water-holding capacity and, therefore, the yield after cooking. Thus, the high water retention capacity attributed to edible mushroom meals makes them excellent substitutes for phosphates. In this regard, different authors [59,60,61] have used edible mushroom flours (*Agaricus bisporus* and *Flammulina velutipe*) as phosphate substitutes in emulsified meat products with very good results. *F. velutipes* meal has also been used as a nitrite substitute in raw-cured ham [60], although in this type of meat product (raw-cured), its application as a phosphate substitute was not satisfactory. On the other hand, different mushroom concentrates have been used as substitutes for monosodium glutamate in the preparation of chicken soups [46], or even the whole mushrooms (*Agaricus bisporus*, steamed or grilled) as binders (replacing egg white) in products such as meatballs or croquettes for vegans [62,63]. In all cases, their technological feasibility and the good sensory acceptance of the resulting foods were the best rated.

Other studies suggest that mushrooms or their extracts can be used as natural preservatives, extending the shelf life of the foods in which they are incorporated, controlling the development of fat oxidation reactions (antioxidants) or inhibiting the growth of micro-organisms involved in their deterioration (antimicrobials). Studies have confirmed this antimicrobial activity in fermented sausages [64] and fresh pork [65], and their antioxidant activity has also been widely described in foods (mainly meat products) in which edible mushrooms were incorporated as substitutes for animal proteins or fats [17,66,67].

## 3. Reduced Salt Meat Products

### 3.1. Functions of Salt in Meat Products

Due to health problems related to excessive salt consumption, many countries have established campaigns to encourage food industries in general and meat industries in particular to develop and market low-salt meat products. In order to produce low-salt or salt-free meat products, it is not enough to eliminate or reduce salt from the formulation. Salt has many functions (technological, health, and sensory) in meat products, and if it is reduced or eliminated, these properties will also be reduced or eliminated, and other actions are needed to mitigate these effects [68].

(a) Technological role of salt in meat products: Salt is essential for the solubilization of meat myofibrillar proteins, which are responsible for the gelling and emulsifying properties of the meat matrix [69]. In addition, salt activates the extraction of meat proteins by improving hydration and water-holding capacity, contributing to improved post-cooking yields of meat products. Other associated effects are increased juiciness and viscosity of meat batters, resulting in the formation of heat-stable emulsions (e.g., frankfurters) [70].
(b) Antimicrobial role of salt in meat products: the antimicrobial activity of salt is based on its ability to reduce the water activity of the meat product, which depends on the amount of salt in the aqueous phase. The presence of salt in the meat matrix can either cause an osmotic shock to the micro-organisms, resulting in their death, or cause serious damage to bacterial cells, resulting in a significant decrease in their multiplication [71]. This property is closely related to the preservation and shelf life of the meat products in which it is incorporated and must be taken into account when reducing the salt content. For example, a study [72] found that reducing salt in bacon from 3.5% to 2.3% decreased shelf life from 56 to 28 days.
(c) Sensory role of salt in meat products: Salt by itself has a very important flavoring effect on the foods in which it is incorporated, although it has also been described as a flavor enhancer. In the case of meat products, the sensory aspects that are affected by salt are not only flavor and aroma but also many textural attributes such as juiciness, cohesiveness, hardness, etc., which are determined by the technological effects mentioned above [68,70].

### 3.2. Strategies to Reduce Salt in Meat Products

**Figure 2:**
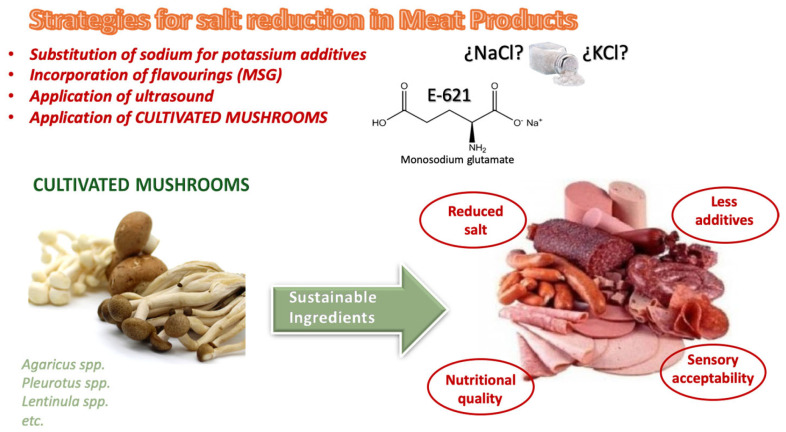
Strategies for salt reduction in meat products.

Several strategies are being developed to reduce salt in meat products (Figure 2), including (i) reformulation of products and (ii) technological developments applied in their processing to reduce the need for its use.

(a) Reformulation of the meat product: Firstly, sodium chloride and other sodium-containing additives typical in the production of cured meat products (such as sodium phosphate, sodium ascorbate, etc.) were replaced by other salts such as potassium chloride or calcium chloride, potassium phosphate, potassium ascorbate, etc. [73]. However, drawbacks such as the appearance of bitter and metallic flavors in the products were reported [74]. To overcome this problem, 50% salt substitutions (using 50% sodium chloride and 50% potassium chloride) combined with the addition of some additives or spices were tested in order to mask these bitter flavors. Some successful studies have been reported, such as the development of a low-salt cured ham (50% salt reduction compared to traditional ham) using potassium lactate as a partial salt substitute [75]. It has also been reported that a balanced combination of potassium and sodium phosphate in the formulation of meat products can effectively reduce the sodium content by 10% and 30% in the finished product without affecting its sensory properties or safety.

Another alternative that has been worked on recently (and on which the application of cultivated mushrooms as salt substitutes is based) is the application of flavorings or flavor enhancers. The most frequently used are yeast extracts, vegetable protein hydrolysates, monosodium glutamate, and 5′-nucleotides. They are used to improve the “savory perception” of products due to umami taste [76]. Umami has been found to have the property of improving the palatability of food by modulating sweetness, enhancing salty taste, and suppressing sour and bitter flavors [77].

In the food industry, glutamic acid and its salt, sodium monoglutamate, have been widely used as the main inducers of the umami taste. However, the use of these flavor-enhancing compounds increases the list of additives in formulations with a consequent rejection by consumers, so new trends are moving toward the use of natural sources of umami-inducing compounds, including certain foods such as meats, cheeses, seafood, vegetables, yeasts, and, especially, mushrooms.

(b) Technological developments make it possible to apply treatments that improve the diffusion of salt in the meat structure so that the same technological functionality can be achieved with a lower salt content without compromising the safety, texture, and flavor of the final product. In this sense, there are very interesting studies on the application of ultrasound [78]. This technology uses sound waves with frequencies higher than those detected by the human ear (>20 kHz). These applications have been performed at intensities higher than 1 W/cm^2^ and at frequencies between 20 and 100 kHz, proving effective in many areas, but especially in protein extraction and inactivation of micro-organisms (by modifications of the permeability of their membranes), two of the most important functions of salt [79]. These effects would accelerate processes such as curing, marinating, and drying meat and meat products, with lower amounts of salt required to achieve the same effects. Many of the parameters involved still need to be optimized for each particular product because it can sometimes increase lipid oxidation and modify sensory characteristics [78], but results on chicken meat products are very promising [80].

Due to the reduction in shelf-life that has been observed in low-salt meat products, the application of high-pressure treatments to these foods has emerged as a useful tool to increase their shelf-life [81]. High pressure is a non-thermal preservation technique based on the application of 300–600 MPas of pressure at moderate temperatures (<45 °C), achieving the preservation of the product but minimally affecting its texture, aroma, appearance, and nutritional value [82]. Some authors have even described a natural increase in salty taste in products treated with high pressure (cured ham and loin). This has been related to changes in the interaction between sodium ions and the structure of meat proteins induced by high pressure. These changes would be responsible for a greater release of sodium from the taste receptors on the tongue, leading to a saltier taste [83].

## 4. Application of Cultivated Mushrooms as Salt Substitutes in Food Production

The main references found about the use of mushrooms in the development of salt-reduced meat products are shown in Table 2. In this table, the species of mushroom used, the type of meat product they were added to, the percentage of salt reduction achieved, and the main properties affected in the resulting product are described.

Regarding the studies on beef meat products, Myrdal–Miller et al. [84] and Guinard et al. [40] used *Agaricus bisporus* in beef taco filling, not only for the partial reduction of salt (25%) but also for meat reduction, reporting improvements in the aroma and flavor of these fillings when mushrooms were incorporated. This same mushroom was also used by Wong et al. [45,85] for a 45% salt reduction in the meat mixture used for taco fillings and a 25% salt reduction in beef patties, respectively. Salt reductions of up to 50% have been reported using *Agaricus bisporus* and *Pleorotus ostreatus* in beef patties [49]. Similar salt reduction percentages were achieved in the production of beef patties with the use of 20% aqueous extract of *Lentinula edodes* [86]. Recently, using flours obtained from oyster mushrooms (*Pleurotus ostreatus*), button mushrooms (*Agaricus bisporus*), and portobello mushrooms (*Agaricus brunnescen*), salt reduction percentages even higher than 50% (55–61%) have been successfully achieved in beef burgers [87]. The authors reported that despite having certain undesirable effects on cooking properties, texture, and color, the final beef burgers were not negatively valued by consumers.

In the case of chicken products, salt reductions of up to 50% have been reported using mushrooms (champignon at 5.7%) in a dish prepared with chicken meat (chicken stroganoff) [88]. In some cases, although the flavoring properties of mushrooms (or their extracts) have been sufficient to improve the taste and increase the perception of “saltiness” in meat products produced with reduced salt content, even to the extent of detecting no sensory differences in this attribute compared to the original meat products (without salt reduction), modifications in the technological properties of these meat products have been described. For example, Akesowan and Jariyawaranugoon [74] used fresh *Agaricus bisporus* (20%) with the intention of being able to reduce the salt content of chicken nuggets by up to 45%, had to reduce their expectations to a feasible reduction of only 13% salt, because of the effects of lower salt proportions on important technological properties such as cooking losses, shrinkage, and firmness of the nuggets. Other authors have also used cultivated mushrooms, not as salt substitutes per se, but to improve the quality of low-salt meat products. Jo et al. [60] incorporated freeze-dried edible winter mushrooms (*Flammulina velutipes*) (5–10 g/kg) in the formulation of low-salt chicken sausages (3 g/kg), improving their nutritional quality (higher dietary fiber content) and resistance to lipid oxidation, reducing the amount of exuded rendered fat, without negatively affecting their sensory properties.

In the case of pork meat products, salt reductions of up to 50% have been reported using *Agaricus bisporus* and *Pleorotus ostreatus* in cooked meat products such as Frankfurter sausages or pâté [48,89]. In both cases, the use of *A. bisporus* resulted in darker products, while *P. ostreatus* showed a higher impact on the texture. Nevertheless, the final products were sensorialy acceptable, and the substitution did not affect their shelf-life.

**Table 2:** Application of edible mushrooms in the development of low-salt meat products.

Mushroom Species	Meat Product	Reduction of Salt	Features Affected	Reference
*A. bisporus*	Mixed beef for tacos	25%	Improved aroma and flavor	[84]
*A. bisporus*	Taco meat mix	25%	Improved aroma and flavor	[40]
*A. bisporus*/*P. ostreatus*	Frankfurter sausages	50%	Color (dark) Sensory acceptable	[48]
*A. bisporus*/*P. ostreatus*	Beef burger	50%	Changes in texture and color Sensory acceptable	[49]
*A. bisporus*/*P. ostreatus*	Pâté	50%	Sensory acceptable Improved texture Color (dark)	[89]
*A. bisporus*	Taco meat mix	45%	Improved aroma and flavor	[45]
*F. velutipes*	Chicken sausages	25%	Delays lipid oxidation Textural changes No change in color or sensory properties	[60]
*A. bisporus*	Chicken nuggets	25 and 50%	Improved aroma and flavor Improved textural changes due to salt reduction	[74]
*L. edodes*	Beef burger	50%	Improved aroma and flavor	[86]
*A. bisporus*	Beef burger	38 and 75%	Sensory modifications at high % substitution Retards oxidation	[50]
*P. ostreatus*/*A. bisporus*/*A. brunnescen*	Beef burger	55 and 61%	Increased cooking loss and shrinkage Sensory acceptable	[87]

All these studies confirmed that the ability of cultivated mushrooms to be used as salt substitutes in meat products depends not only on the species used but also on the cultivation and the way they are applied. These factors undoubtedly influence the higher or lower content of the compounds responsible for the umami taste and, therefore, determine their suitability for such purposes (salt reduction). Studies have also shown that the technological processes used to prepare the ’ingredient’ itself from the selected mushrooms are a determining factor in terms of their effect on the content of the compounds responsible for the umami taste. For example, the method of drying the mushrooms (air drying, hot air drying, vacuum drying, or freeze-drying) seems to be quite relevant [90,91].

Moreover, this field is going even one step further to contribute to the sustainability of food production and the food industry. As we have already seen the usefulness of cultivated mushrooms in the production of low-salt meat products, it has been proposed to use not the mushrooms themselves, but the co-products generated from their cultivation, namely the mushroom stems. Studies report considerable amounts of umami compounds in mushroom stems, i.e., the parts that remain after cutting or harvesting [92,93]. Harada-Palermo et al. [93] developed an “umami ingredient” from the stems of shiitake mushrooms and successfully applied it in the production of low-sodium beef patties [94]. The application of this “umami ingredient” in burgers did not affect the pH, water activity, or color of the burgers and exerted positive effects on cooking properties and texture.

## 5. Conclusions

Cultivated mushrooms have a high nutritional value, are a source of protein and dietary fiber, vitamins, minerals, and bioactive compounds, and are also low in fat, sodium, and calories. They are natural products whose production is economical and sustainable. These characteristics make mushrooms an ideal ingredient (natural origin, from sustainable production, with a nutritious and healthy composition, with useful technological properties and minimally affecting the sensory properties of the reformulated products) for application in the development of healthier and more sustainable foods. Their content of compounds responsible for the umami taste also makes them an ideal ingredient for improving the quality of reduced salt meat products. The potential of this aspect of mushrooms in the meat industry is immense, and their application is recent but already has very promising results (50% salt substitution has been achieved), especially in fresh meat products (hamburgers) and heat-treated meat products (sausages, pâté, roast meat, etc.). However, due to the variability in the composition of mushrooms depending on the species, area, and method of cultivation, treatment, preservation, etc., it is necessary to optimize the processing conditions for each of the meat products in which it is applied. Further research and optimization are needed to minimize changes in product color and texture, ensuring high consumer acceptance of mushroom-based, low-sodium meat products.

## Figures and Tables

**Table 1:** Chemical composition (g/100 g) of the most widely consumed cultivated mushrooms in Spain.

	Water	Proteins	Lipids	Carbohydrates	Fiber
*A. bisporus*	91.4	1.4	0.2	3.2	2.0
*P. ostreatus*	88.8	3.2	0.3	5.4	1.5
*A. brunnescens*	91.5	2.7	0.3	4.7	2.1
*L. edodes*	88.6	2.4	0.2	6.8	2.5

Kalac [23]; Tolera and Abera [24]; Dimopoulou et al. [25]; USDA [26].

## Data Availability

No new data were created or analyzed in this study. Data sharing is not applicable to this article.
